# Cure of Cancer by Erysipelas

**Published:** 1890-08-09

**Authors:** 


### CURE OF CANCER BY ERYSIPELAS.

That malignant growths have diminished or disappeared after attacks of erysipelas has long been known, and now that the coccus of erysipelas has been determined and been shown to be capable of artificial cultivation, the question arises whether inoculations therewith might not be justifiably employed in the treatment of carcinomatous, sarcomatous, and other neoplasms, when circumstances render their removal by the knife impracticable. Dr. Kleeblatt reports three cases, the results of two of which were very satisfactory. In one, a case of lymphosarcoma, a tumour in the tonsil had been removed, but numerous others appeared subsequently in the carotid glands and suprascapular fossa varying in size from a hen's egg to that of an infant's head. Injections of Fowler's solution gave rise to abscesses, leading to diminution of the tumours, but others appeared in the adjacent regions. After an attack of spontaneous erysipelas with great increase of suppuration there was a marked reduction in size of the tumours, the large one in the supra-scapular fossa, dwindling to the size of a pigeon's egg. Artificial inoculations of erysipelas subsequently led to the complete disappearance of all existing or visible tumours, but another which appeared still later was inoculated unsuccessfully, and the man ultimately died from hæmorrhage consequent on the erosion of the carotid artery. The other two cases were more favourable, cancerous growths involving the lower jaw and nares, suppurated after injections of Fowler's solution, but continued growing; inoculations with erysipelas were followed by the complete disappearance of both tumours which did not return. In the third case, that of a young woman, a lymphadenoma the size of a pigeon's egg under the lower eyelid had been treated with inunctions of Pot. Iod. and Ung. Hydr., which set up some suppuration, but after the occurrence of spontaneous erysipelas the tumour disappeared in the course of six days.

